# A cross-syndrome cohort comparison of sleep disturbance in children with Smith-Magenis syndrome, Angelman syndrome, autism spectrum disorder and tuberous sclerosis complex

**DOI:** 10.1186/s11689-018-9226-0

**Published:** 2018-03-01

**Authors:** J. Trickett, M. Heald, C. Oliver, C. Richards

**Affiliations:** 1 Department of Health Sciences, College of Life Sciences, George Davies Centre, University of Leicester, Leicester, LE1 7RH UK; 2 Cerebra Centre for Neurodevelopmental Disorders, School of Psychology, University of Birmingham, Birmingham, B15 2TT UK

**Keywords:** Sleep disturbance, Cross-group comparison, Intellectual disability, Genetic syndromes, Angelman syndrome, Smith-Magenis syndrome, Tuberous sclerosis complex, Autism spectrum disorder

## Abstract

**Background:**

Sleep disturbance is common in children with neurodevelopmental disorders, with high rates identified in children with Smith-Magenis syndrome (SMS), Angelman syndrome (AS), autism spectrum disorder (ASD) and tuberous sclerosis complex (TSC). Phenotypic sleep profiles for these groups may implicate different pathways to sleep disturbance. At present, cross-group comparisons that might elucidate putative phenotypic sleep characteristics are limited by measurement differences between studies. In this study, a standardised questionnaire was administered across groups affording comparison of the prevalence and profile of sleep disturbance between groups and contrast to chronologically age-matched typically developing (TD) peers.

**Methods:**

The modified version of Simonds and Parraga’s sleep questionnaire, adapted for use in children with intellectual disabilities, was employed to assess sleep disturbance profiles in children aged 2–15 years with SMS (*n* = 26), AS (*n* = 70), ASD (*n* = 30), TSC (*n* = 20) and a TD contrast group (*n* = 47). Associations between sleep disturbance and age, obesity, health conditions and overactivity/impulsivity were explored for each neurodevelopmental disorder group.

**Results:**

Children with SMS displayed severe night waking (81%) and early morning waking (73%). In contrast, children with ASD experienced difficulties with sleep onset (30%) and sleep maintenance (43%). Fewer children with ASD (43%) and AS (46%) experienced severe night waking compared to children with SMS (both *p* < .01). Higher sleep-disordered breathing scores were identified for children with SMS (*p* < .001) and AS (*p* < .001) compared to the TD group. Sleep disturbance in children with AS and TSC was associated with poorer health. Children experiencing symptoms indicative of gastro-oesophageal reflux had significantly higher sleep-disordered breathing scores in the AS, SMS and ASD groups (all *p* < .01). A number of associations between overactivity, impulsivity, gastro-oesophageal reflux, age and sleep disturbance were found for certain groups.

**Conclusions:**

These data reveal syndrome-specific profiles of sleep disturbance. The divergent associations between sleep parameters and person characteristics, specifically symptoms of gastro-oesophageal reflux, overactivity and impulsivity and age, implicate aetiology-specific mechanisms underpinning sleep disturbance. The differences in prevalence, severity and mechanisms implicated in sleep disturbance between groups support a syndrome-sensitive approach to assessment and treatment of sleep disturbance in children with neurodevelopmental disorders.

**Electronic supplementary material:**

The online version of this article (10.1186/s11689-018-9226-0) contains supplementary material, which is available to authorized users.

## Background

Sleep disturbance is common in children with neurodevelopmental disorders [1] and associated with deleterious outcomes for individuals and families, including elevated levels of difficult behaviour and increased parental irritability and fatigue [2, 3]. A recent systematic review outlining the prevalence of sleep disturbance revealed that children with Smith-Magenis syndrome (SMS) and Angelman syndrome (AS) evidenced the highest rates of sleep disturbance (100 and 48–70% respectively) compared to children with other rare genetic syndromes [1]. Similarly, children with autism spectrum disorder (ASD) and tuberous sclerosis complex (TSC) are reported to show strikingly high rates of sleep disturbance (ASD 50–80% [4], TSC 74% [5]). Despite these elevated prevalence rates, there is limited research examining the *profile* of sleep disturbance across these neurodevelopmental disorders, specifically the relative contributions of settling problems, night waking and early morning waking alongside parasomnias, sleep anxiety and sleep-disordered breathing to the presentation of sleep disturbance in each group. Thus, there is a need to contrast profiles of sleep disturbance in children with a range of neurodevelopmental disorders to evaluate characteristics of sleep disturbance that are more common in a phenotype but are shared by other phenotypes [6]. The emergence of divergent profiles would suggest that aetiology of neurodevelopmental disorder, specifically the phenotypic characteristics of each neurodevelopmental disorder, is important in assessment and intervention for sleep disturbances.

Within behavioural phenotype research, there is a debate as to the most appropriate study design to assess the partial specificity of behavioural characteristics [6], with studies employing within and between syndrome designs, and comparisons to mental or chronological age-matched typically developing peers. The inclusion of a typically developing contrast group may be particularly important in sleep research as sleep is a universal state experienced by mammals, entrained by the body’s circadian rhythm, and hypothesised to consolidate learning, restore cells and synaptic plasticity [7]. Sleep, therefore, is a necessary health consideration for children regardless of disability. To understand the degree of impairment in children with neurodevelopmental disorders, it is necessary to consider what is ‘typical’ in an aspect of health which has considerable interpersonal variability [8]. Importantly, sleep disturbance in children with neurodevelopmental disorders is hypothesised to be qualitatively commensurate to that experienced by typically developing children, but more pervasive and persistent [9].

The limited studies that have evaluated the profile of sleep disturbance in SMS, AS, ASD and TSC compared to TD children reveal some important syndrome-related patterns. In SMS, shorter total sleep time, earlier bedtimes, earlier final morning wake times and an inverted circadian rhythm have been identified in an actigraphy study of eight children with SMS compared with age-matched TD children [10]. A wide range of sleep disturbances were found in a questionnaire study including individuals with AS, namely overall shorter sleep duration characterised by significantly longer sleep onset latency, more frequent wakings with difficulty falling back to sleep, significantly more frequent unusual movements and jerking during sleep, sleep-disordered breathing and more frequent daytime somnolence compared with age-matched TD individuals [11]. In children with TSC, shorter total sleep time, as assessed by polysomnography and the modified Simonds and Parraga sleep questionnaire [12], is attributed to specific problems with settling at sleep onset and night waking compared with TD children [5, 13]. Sleep disturbance in children with ASD may be differentiated from that of other neurodevelopmental disorder groups by greater bedtime resistance and sleep anxiety compared to TD children, in addition to higher scores on questionnaire measures of sleep-disordered breathing, sleep onset delay, sleep duration and night waking [14]. There has been substantial characterisation of sleep disturbance in children with ASD, but comparatively little evaluation of sleep parameters in AS, SMS and TSC despite similar or higher prevalence of sleep disturbance in these groups. Much of the existing data are limited by small samples, lack of validated questionnaires, omission of the study of parasomnias and the lack of comparison with other neurodevelopmental disorder groups. Thus, it is not possible to draw comparisons between the groups to evaluate the potential for partial specificity as differing definitions of sleep disturbance were used between studies. Therefore, further research utilising a standardised measure of sleep disturbance across these groups is warranted.

In addition to contrasting the profile of sleep disturbance between neurodevelopmental groups, there is also a need to elucidate associations between sleep parameters and comorbid health and behaviour difficulties, in addition to demographic variables such as age and verbal ability. These contrasts will delineate whether patterns of comorbidities with sleep disturbance are shared or divergent across children with different neurodevelopmental disorders and may allude to syndrome-related pathways to sleep disturbance.

A number of health conditions with heightened prevalence in children with neurodevelopmental disorders such as epilepsy and gastro-oesophageal reflux have been associated with sleep disturbance in typically developing and clinical populations [15, 16]. Specifically, in AS, in which seizures affect 80% of individuals [17], the presence of multiple types of seizures and refractory epilepsy (77% of individuals) have both been associated with parent-reported sleep disturbance [18, 19]. However, two large-scale studies [20, 21] have failed to replicate the association between seizures and sleep quality in individuals with AS perhaps due to methodological differences. Seizures affect 70% of individuals with TSC [22]. Two studies have reported a higher prevalence of night waking in children with TSC who experienced seizures in the last 6 months or who had three or more nightly seizures [5, 13]. The contribution of anti-epilepsy drugs (AEDs) to sleep quality requires consideration, as AEDs have been found to both prolong total sleep time or induce daytime sleepiness and disturb sleep quality or onset according to self-report in adults with TSC [23]. Seizures also affect up to 18% of children with SMS [24] and 30% of children with ASD [25], with seizures associated with sleep disturbance in children with ASD [26]. The putative associations between sleep quality and epilepsy need elucidating further to ensure that appropriate assessment protocols and interventions to address any neurological aetiology of sleep disturbance are developed.

In addition to epilepsy, children with SMS, AS, TSC and ASD are also more likely to experience painful health conditions than typically developing children. These vary in aetiology between syndrome groups with multi-organ tumours common in TSC, chronic otitis media affecting children with SMS, scoliosis commonly occurring in children with SMS and AS, and high relative risk of gastrointestinal symptoms, asthma and allergies in children with ASD [27–30]. The presence of these health conditions is associated with sleep disturbance. For example, obstructive sleep apnoea has been associated with gastro-oesophageal reflux in children without an intellectual disability [16]; similarly, children with an intellectual disability who experience pain were more likely to snore and have sleep-disordered breathing problems [31]. In children with ASD, those with gastrointestinal problems and/or epilepsy were twice as likely to have sleep problems [32]. Pain has also been associated with shorter sleep duration, more parasomnias and sleep-disordered breathing in ASD [33]. Despite the high prevalence of sleep disturbance *and* physical health conditions, no studies have considered the putative associations between pain or gastro-oesophageal reflux and sleep in children with AS, SMS and TSC.

Alongside painful health conditions, other adverse health outcomes associated with poor sleep in the paediatric population should also be evaluated in children with neurodevelopmental disorders. Obesity is identified as an independent risk factor for both snoring and obstructive sleep apnoea in typically developing children [34]. Obesity has been suggested as a risk factor for obstructive sleep apnoea in children with SMS [35], and children with AS may be at heightened risk of obesity due to purported impaired satiety and behaviours such as taking and storing food [36]. Obesity may mediate the relationship between obstructive sleep apnoea and daytime sleepiness, as described in typically developing children [37], and requires evaluation in these neurodevelopmental groups.

In summary, examining the putative associations between sleep and painful health conditions, health problems such as epilepsy and adverse health outcomes such as obesity will provide clinicians with important information about comorbidities between health and sleep in children with neurodevelopmental disorders. Describing these associations between neurodevelopmental disorders will inform syndrome-sensitive models of the development and maintenance of sleep disturbance in these groups.

Finally, in contrast to physical health problems, there are also demographic and behavioural characteristics purported to be associated with sleep disturbance. In the typically developing population and in children with attention deficit and hyperactivity disorder (ADHD), hyperactivity is associated with daytime sleepiness [38, 39], and in turn, hyperactivity and daytime sleepiness are associated with sleep-disordered breathing [34, 40]. However, the relationship between ADHD characteristics, daytime sleepiness and sleep-disordered breathing has not been evaluated in children with neurodevelopmental disorders, despite a clinical imperative associating ADHD characteristics and parent stress in these groups [41].

A greater proportion of younger children experience sleep problems than primary school-aged children in the typically developing population [42, 43]. A meta-analysis of sleep quality in children with intellectual disabilities demonstrated that age was not significantly related to impaired sleep quality; however, as these data are cross-sectional, they should be interpreted with caution [44]. The persistence of sleep problems across the developmental trajectory of AS is unclear, as some cross-sectional and longitudinal studies in small samples have shown a reduction in the prevalence of sleep problems after the age of 6 [45, 46]. However, other studies using age as a correlate or comparing between arbitrary age groups have suggested that sleep difficulties persist with age [11, 21]. Increasing age is associated with more disturbed sleep in SMS [47], whilst for children with ASD, a shortened sleep duration is observed from the age of 30 months relative to typically developing peers [48]. The relationship between age and sleep quality has not been examined in children with TSC. These findings suggest that the relationship between age and sleep quality in children with specific syndromes needs to be examined further. Poorer receptive and expressive language skills have been associated with poorer sleep quality [49]. The relationship between children’s verbal ability and sleep quality, particularly concerning children’s ability to make their needs known during the night, is an area that requires further research.

In summary, there is a need to describe the prevalence and profile of sleep disturbance across neurodevelopmental disorders in order to evaluate the evidence for partial specificity of sleep disturbance profiles related to phenotypic characteristics. Additionally, there is a need to evaluate the putative associations between sleep disturbance and children’s health, overactivity and impulsivity, age and ability in each group to determine the likelihood of syndrome-specific pathways to sleep disturbance. The use of a typically developing contrast group will enable the comparative severity of sleep disturbance in each neurodevelopmental group to be quantified relative to age-expected norms. Accounting for the variation in degree of intellectual disability both within and between disorder groups will ensure that sleep disturbance specificity is accounted for beyond associations with severity of intellectual disability. Therefore, the present study has the following aims:

1. To describe the profile of sleep disturbance in children with SMS, AS, TSC and ASD compared to typically developing children and to compare sleep disturbance profiles between different neurodevelopmental disorder groups
2. To evaluate the nature of the relationship between age and sleep disturbance for each neurodevelopmental disorder group
3. To describe associations between verbal ability, behavioural (overactivity and impulsivity), health (presence of seizures, severity of health problems, presence of symptoms of gastro-oesophageal reflux) and sleep disturbance variables for each neurodevelopmental disorder group
4. To explore the association between sleep-disordered breathing and obesity for each neurodevelopmental disorder group
5. To examine the relationship between daytime sleepiness and sleep-disordered breathing

## Methods

### Participants

Participants with AS, SMS, ASD and TSC were recruited via the Angelman syndrome UK family support group (ASSERT), the Foundation for Angelman Syndrome Therapeutics in the USA, the Smith-Magenis Syndrome Foundation UK, Autism West Midlands, Cerebra, the Tuberous Sclerosis Association and existing databases held by the Cerebra Centre for Neurodevelopmental Disorders at the University of Birmingham. A community sample of typically developing children was recruited via friends and family of researchers at the Cerebra Centre for Neurodevelopmental Disorders. Typically developing children were excluded if they held a statement of education need that indicated an additional need that may influence sleep, for example, ADHD and hemiplegia. Demographic characteristics for the groups are presented in Table 1.

**Table 1:** Demographics of each group

		AS (*n* = 70)	SMS (*n* = 26)	TSC (*n* = 20)	ASD (*n* = 30)	TD (*n* = 47)	*χ* ^2^	df	*p* value	Post hoc analysis (*p* < .01)
Mean age (SD)		8.64 (3.77)	8.54 (3.08)	7.20 (4.11)	9.45 (3.47)	8.00 (3.24)	5.66	4	.226	–
Males (%)		45.7	61.5	55.0	70.0	66.0	7.50	4	.112	–
Genetic mechanism (%)	Deletion	70.0	92.3	–	–					
	Mutation	7.1	0	–	–					
	TSC1	–	–	15.0	–					
	TSC2	–	–	60.0	–					
	UPD	11.4	0	–	–					
	Imprinting	7.1	0	–	–					
	Other	1.4	0	5.0	–					
	Translocation	1.4	3.8	–	–					
	Clinical diagnosis	1.4	0	5.0	100.0					
	Unknown	0	3.8	0	0					
	Awaiting genetic results	0	0	5.0	–					
	Missing data			10.0						
BMI category^a^	Overweight (%)	28.9	25.0	26.3	13.0	–	1.76	4	.779	–
Speech	Verbal (%)	8.6	84.6	80.0	76.7	100	120.88	4	< .001	TD, ASD, TSC; SMS > AS
Mobility	Walk unaided (%)	72.9	100	100	96.7	100	41.31	4	< .001	TD, ASD, SMS; TSC > AS
Currently/occasionally using melatonin or other sleep medication (%)		71.4	46.2	30.0	40.0	2.1	53.52	4	< .001	AS > ASD; TSC, ASD, SMS > TD
Symptoms indicative of reflux (%)		67.1^b^	64.0^c^	35.0	43.3	–	9.68	3	.021	AS > ASD, TSC
Seizures last month (%)		62.9	11.5	80.0	3.3	–	30.21	3	< .001	TSC > ASD, SMS; AS > ASD, SMS
Taking anti-epilepsy medication (%)		70.0	3.8	75.0	0	–	65.79	3	< .001	TSC > ASD, SMS; AS > SMS, ASD
TAQ impulsivity mean (SD)		15.25^b^ (5.19)	17.92 (2.65)	13.30 (7.17)	15.47 (4.92)	–	7.28	3	.064	–
TAQ overactivity mean (SD)		17.40^b^ (9.16)	21.0^c^ (7.25)	16.0 (10.99)	21.57 (15.92)	–	5.01	3	.171	–

^a^Data related to 64 children with AS, 15 children with SMS, 19 children with ASD and 18 children with TSC

^b^Data only related to 30 children with AS

^c^Data only related to 25 children with SMS

The data in Table 1 demonstrate that significantly fewer children with AS had 30 or more words or signs in their vocabulary compared to children with ASD, SMS or TSC. Significantly more children with AS and TSC experienced seizures in the last month compared with children with ASD and SMS. A significantly greater proportion of children with AS were unable to walk unaided or lacked verbal language compared with all other groups. Seventy one percent of children with AS used sleep medication, compared with only 40% of children with ASD and 30% of children with TSC. No other significant differences were found between groups.

### Procedure

Questionnaires were hosted via limeSurvey, and the information sheet, consent form and questionnaires were accessed via a password-protected URL link. Paper copies of questionnaires were also made available. Families were sent a link via email or by letter or accessed the link via a syndrome support group Facebook page, the Cerebra Centre for Neurodevelopmental Disorders Facebook page or website.

### Materials

The following questionnaires were administered. A demographic questionnaire was included to obtain data pertaining to the child’s age and diagnosis and verbal ability (does your child have more than 30 words or signs in their vocabulary?) and whether their child is able to walk unaided.

The Health Questionnaire [50] was administered to assess the severity of any current (i.e. experienced within the last month) medical condition. Severity was measured using a 4-point Likert scale (never, mild, moderate or severe). The authors report good inter-rater reliability for this questionnaire, with a mean Kappa value of .65 for the overall health problem score. Additional questions to assess medication usage were included.

The Activity Questionnaire (TAQ) [51] was administered to assess the six aspects of hyperactivity and three aspects of impulsivity described in the DSM IV. The overactivity and impulsivity subscale scores were calculated according to the authors’ guidelines [52], and in line with the recommendations, the impulsivity subscale was pro-rated for immobile participants [52]. A 5-point Likert scale measures the frequency of overactive and impulsive behaviours over the last month, ranging from ‘never/almost never’ to ‘always/almost all of the time’. Inter-rater reliability is good for both the impulsivity and overactivity subscales (.74 and .70 respectively). Test-retest reliability is also good for the impulsivity and overactivity subscales (.88 and .87 respectively) [52].

The modified version of Simonds and Parraga’s sleep questionnaire (MSPSQ) [12, 53] was administered to assess sleep disturbance in the groups. The MSPSQ has been validated for use with individuals aged 2 to 16 with an autism spectrum disorder to include children with an intellectual disability [53]. Questions address bedtime routine and any severe settling, early morning waking or night waking problems (that occur at least three times a week, taking over an hour to fall asleep, waking before 5 am or wakings that last over a few minutes) [54] as well as any treatment that parents may have tried for their child’s sleep and whether or not they considered the treatment to be effective. The questionnaire assesses the time of sleep onset and timings of nocturnal and daytime sleep and sleep latency over the last month. A confirmatory factor analysis revealed five domains to the MSPSQ: snoring, daytime sleepiness, complaints related to sleep, sleep apnoea and anxiety related to sleep [55]. The questionnaire has adequate internal consistency (α = .67) with total sleep quality scores correlating strongly with the total scores on the widely used Child Sleep Habits Questionnaire [53]. Questions about children’s height and weight for BMI analysis were also obtained from the MSPSQ. BMI scores were calculated by converting all measurements to metric and using the equation weight (kg)/height^2^ (m). BMI percentiles were derived using the Childhood and Puberty Close Monitoring chart developed by the Royal College of Paediatrics and Child Health for use with children with possible growth or nutritional problems and recommended for use in special schools [56]. Children with a BMI percentile of 91 or above were classified as overweight as per the chart’s guidelines. For the purpose of the analysis, children classed as overweight were compared to children with a healthy weight (children classed as underweight were excluded).

The *Gastro-oesophageal Distress Questionnaire* (*GDQ*) [57] was administered to assess behaviours indicative of gastro-oesophageal reflux. The GDQ measures the frequency of a range of possible reflux symptoms on a 5-point Likert scale ranging from ‘does not occur’ to ‘more than once an hour’. The 17 items were assessed over the last 2 weeks. Scores indicating the presence of five daily occurring symptoms of possible gastro-oesophageal reflux were considered to be clinically significant. Question 14 pertaining to night waking was removed to ensure the independence of variables when comparisons were made with sleep parameters.

### Analysis

Data were tested for normality, and several variables were found to be non-normally distributed; therefore, non-parametric statistical tests were used. Differences between groups for demographic variables were assessed using chi-squared analyses. The MSPSQ subscale scores and the portion of children who experience severe sleep disturbances derived from the MSPSQ (nocturnal waking, early morning waking, difficulty settling/initiating sleep) were compared between groups using chi-squared or Kruskal-Wallis tests. Post hoc Mann-Whitney *U* tests were used to identify the significant between group differences for continuous variables.

Effect sizes were evaluated using Cohen’s *r* (*z*/square root of *N*) which is equivalent to Cohen’s *d* for non-parametric tests. In light of the lack of literature using effect sizes to evaluate sleep disturbance in children with neurodevelopmental disorders, guidelines to interpret Cohen’s *r* using values outlined by Cohen [58] were used (.2 = small effect, .5 = medium effect, .8 = large effect [59]).

Odds ratios were used for nominal data and arbitrary cut-offs for illustration in Table 6. To reduce type I error, an alpha level of *p* < .01 was applied.

Spearman correlations were used to explore the relationships between age, health, overactivity, impulsivity and MSPSQ scores. Spearman correlations were also used to explore the relationships between overactivity/impulsivity and daytime drowsiness/sleep-disordered breathing. Due to the lack of independence between the overactivity subscale and the daytime sleepiness subscale, which contained an item about whether children were more active during the day than other children, only the drowsiness item “seems drowsy but can stop themselves falling asleep” was included in this analysis. Cronbach’s alphas were calculated for the daytime sleepiness subscale for each neurodevelopmental disorder group. Cronbach’s alpha scores for each group averaged .20 (range − .09 to .38). The published psychometrics for the MSPSQ also reveal poor internal consistency for the daytime sleepiness subscale. Despite the poor internal consistency, the use of one item, drowsiness, was chosen to represent daytime sleepiness as the lack of independence of the more active item would have undermined the validity of the analysis.

Mann-Whitney *U* tests were used to examine the putative association between verbal ability, presence of seizures (AS and TSC groups only due to small subsamples of children with SMS and ASD experiencing seizures; see Table 1) and meeting cut-off score for symptoms indicative of gastro-oesophageal reflux and differences in sleep questionnaire subscale scores. A Kruskal-Wallis test was conducted to compare MSPSQ subscales between the genotypes of AS.

## Results

### Relationship between sleep medication, epilepsy medication use and sleep disturbance for each neurodevelopmental disorder group

The demographic information in Table 1 reveals that significantly more children in the AS group took medication to improve their sleep quality than children with ASD and TSC. Children with ASD who took medication to improve sleep quality had significantly higher scores on the night waking subscale (*U* = 31.5, *p* = .001) than children who did not take sleep medication. No other significant differences in any of the other groups were found between children who did and did not take sleep or epilepsy medication. Therefore, all further analyses were conducted combining those who did and did not take medication to improve their sleep.

### Comparing sleep between children with neurodevelopmental disorders and typically developing children (aim 1)

The first aim of the study was to compare sleep disturbance in children with each neurodevelopmental disorder to that of typically developing children and to contrast sleep between neurodevelopmental groups. The results of the comparisons between each neurodevelopmental group and the TD contrast group are presented in Tables 2 and 3.

**Table 2:** Effect sizes for MSPSQ comparisons for each neurodevelopmental disorder group compared to the TD group

	AS–TD	SMS–TD	TSC–TD	ASD–TD
Sleep anxiety^a^	–	− .09	− .01	− .18
Bedtime resistance^b^	− .07	− .19	− .12	− .27^†^
Sleep onset latency	− .11	− .11	− .22^†^	− .59**
Night waking^b^	− .60**	− .76**	− .35*	− .49*
Sleep-disordered breathing^b^	− .47*	− .68**	− .17	− .30^†^
Parasomnia^b^	− .45*	− .64**	− .38*	− .46*
Daytime sleepiness	− .60**	− .73**	− .40*	− .44*
Drowsy during the day	− .49*	− .83**	− .36*	− .38*
Co-sleeping^c^	− .29^†^	− .22^†^	− .35*	− .20^†^

^a^Due to the majority of items on the sleep anxiety subscale requiring verbal ability, this subscale score was not calculated for children with AS

^b^Missing data. Children with ASD: sleep anxiety, bedtime resistance, sleep-disordered breathing and parasomnias (*n* = 29). Children with SMS: bedtime resistance (*n* = 25), sleep-disordered breathing and parasomnias (*n* = 24). TD children: night waking (*n* = 43), sleep-disordered breathing (*n* = 44), sleep onset latency (*n* = 45), parasomnias (*n* = 46)

^c^Co-sleeping item was not administered to 40 children with AS, *n* = 30 for this item

*r ≥ .2—small effect, **r ≥ .5—medium effect (Cohen, [58]), ^**†**^non-significant effect size, *p* > .01

**Table 3:** Odds ratios for MSPSQ comparisons for each neurodevelopmental disorder group compared to the TD group

	AS–TD	SMS–TD	TSC–TD	ASD–TD
Severe night waking problems	18.95 (4.26–84.27)	94.50 (16.92–527.57)	18.41 (3.47–97.60)	17.21 (3.51–84.36)
Severe early morning waking problems	10.49 (1.32–83.20)	124.86 (14.37–1085.24)	8.12 (.79–83.48)	14.0 (1.62–120.70)
Severe settling problems	1.01 (.16–6.27)	1.88 (.25–14.16)	3.97 (.61–25.87)	9.64 (1.91–48.60)

The data in Table 2 demonstrate that all neurodevelopmental disorder groups had significantly higher scores on the night waking, daytime sleepiness and the parasomnia subscales in comparison with the TD contrast group. Medium-sized effects were evident for parasomnia scores in the SMS group and for daytime sleepiness scores for both the SMS and AS groups.

Children with AS and SMS obtained significantly higher night waking scores compared to the TD contrast group, with medium effect sizes. Children with SMS, AS and ASD (trend only) had significantly higher scores on the sleep-disordered breathing subscale compared with the TD contrast group (small effect sizes for AS and ASD groups, medium effect size for the SMS group). A significantly higher score on the sleep onset latency subscale was found for children with ASD compared with the TD contrast group (medium effect size), whilst higher scores on the sleep onset latency subscale for the TSC versus the TD group represented a small non-significant effect size. The data in Table 4 show the profiles of sleep disturbance across children with ASD, TSC, SMS and AS.

**Table 4:** Median (interquartile range) sleep subscale scores, proportions of children with severe sleep problems and statistical comparisons

	AS^a^	SMS	TSC	ASD	TD	Kruskal-Wallis comparison			Between group comparison corrected to *p* < .01
						df	*χ* ^2^	P	
Sleep anxiety	–	11.50 (8.75–13.00)	9.00 (7.25–13.00)	13.00 (9.00–14.50)	10.00 (8.00–13.00)	3	2.90	.408	–
Bedtime resistance	10.00 (7.00–13.00)	14.00 (9.00–16.00)	11.00 (10.0–16.75)	15.00 (9.00–18.00)	11.00 (8.00–14.00)	4	12.85	.012	ASD > AS
Sleep onset latency	2.00 (2.00–3.00)	2.00 (1.00–2.00)	2.50 (2.00–3.00)	3.00 (3.00–5.00)	2.00 (1.00–3.00)	4	36.16	< .001	ASD > SMS, AS, TSC, TD
Night waking	7.00 (5.00–8.00)	7.00 (7.00–8.00)	6.00 (3.25–8.00)	7.00 (4.00–9.00)	3.00 (2.00–5.00)	4	51.72	< .001	ASD, SMS, AS, TSC > TD
Sleep-disordered breathing	9.00 (6.00–11.00)	11.50 (9.00–16.00)	5.00 (5.00–10.75)	7.00 (5.00–11.00)	5.00 (5.00–8.00)	4	40.32	< .001	SMS > ASD, AS, TSC, TD AS > TD
Parasomnia	19.00 (15.00–23.00)	21.50 (18.50–26.75)	19.50 (15.00–23.25)	22.00 (16.00–27.50)	14.50 (11.75–18.00)	4	40.23	< .001	SMS, AS, ASD, TSC > TD
Daytime sleepiness	6.00 (4.00–7.00)	6.00 (6.00–9.00)	6.00 (2.00–6.00)	6.00 (2.00–6.25)	2.00 (2.00–3.00)	4	54.89	< .001	SMS > ASD, TSC, TD AS, ASD, TSC > TD
Drowsy during the day	2.00 (1.00–3.00)^b^	4.50 (3.00–5.00)	1.00 (1.00–2.75)	1.00 (1.00–3.00)	1.00 (1.00–1.00)	4	62.80	< .001	SMS > AS, ASD, TSC, TD AS, ASD, TSC > TD
Co-sleeping	1.5 (1.00–5.00)	1.00 (1.00–4.50)	2.5 (1.00–5.00)	1.00 (1.00–5.00)	1.00 (1.00–2.00)	4	10.58	.032	TSC > TD
Severe night waking (%)	45.70	80.80	45.0	43.30	4.30	4	44.37	< .001	SMS > AS, ASD, TD TSC, AS, ASD > TD
Severe early morning waking (%)	18.60	73.10	15.0	23.30	2.10	4	50.95	< .001	SMS > TSC, AS, ASD, TD AS, ASD > TD
Severe settling (%)	4.30	7.70	15.0	30.0	4.30	4	18.56	.001	ASD > AS, TD

^a^There were no significant differences on any of the MSPSQ subscales between the AS genetic subtypes

^b^Co-sleeping item was not administered to 40 children with AS, *n* = 30 for this item

Children with TSC co-slept with another person at sleep onset or during the night significantly more frequently than TD children, and a trend towards significance was found for children with AS relative to TD children (*p* = .011; see Additional file 1: Table S1). A significantly greater proportion of children with SMS had severe early morning waking problems compared to all other groups and severe night waking problems compared children in the ASD, AS and TD groups. Children with SMS had significantly higher sleep-disordered breathing scores than children in all other groups (*p* < .01) and significantly higher daytime sleepiness scores compared to children with TSC and ASD (*p* < .01, see Additional file 1: Table S1).

Significantly more children with ASD had severe settling problems than children with AS (*p* < .001), and children with ASD also had significantly higher bedtime resistance scores than children with AS (*p* = .002). Children with ASD had significantly higher sleep onset latency scores than all other groups (*p* < .01, see Additional file 1: Table S1).

### Relationships between sleep disturbance and age (aim 2)

There was a significant negative correlation between age and night waking scores for children with AS; hence, night waking appears to decrease with age (*r*_*s*_ = −.32 *p* = .008). There were no other significant relationships between age and sleep quality for children with AS, SMS, TSC, ASD or the TD contrast group.

### Relationships between children’s sleep disturbance, verbal ability, health conditions, epilepsy and behaviour (aim 3)

#### Relationships between sleep disturbance and verbal ability

Children with ASD classed as ‘non-verbal’ (having less than 30 words or signs in their vocabulary) were more likely to experience severe early waking problems (57.1%) compared with children classed as verbal (13.0%, trend towards significance *χ*^2^ = 5.83, *p* = .016). There were no significant differences in the proportion of verbal and non-verbal children who experienced severe sleep problems for any of the neurodevelopmental disorder groups.

#### Relationships between sleep disturbance and the severity of health conditions

Higher sleep-disordered breathing (*r*_*s*_ = .60, *p* < .001) and parasomnias scores (*r*_*s*_ = .36, *p* = .05, trend only) were related to the severity and number of heath conditions experienced by children with AS. Higher night waking (*r*_*s*_ = .54, *p* = .013, trend only), sleep-disordered breathing (*r*_*s*_ = .62, *p* = .004) and parasomnias (r_s_ = .63, *p* = .003) were all positively related to the severity and frequency of health conditions in children with TSC. There were no other associations between sleep subscales and severity of health conditions for any other groups.

#### Relationships between sleep disturbance and severity of epilepsy

Due to the very small numbers of children who had experienced seizures or neurological referrals in the last month in the SMS and ASD groups, these groups were not included in this analysis. There were no significant differences in any of the sleep subscale scores between children who had or had not experienced seizures/neurological referrals in the last month for children in the AS and TSC groups.

#### Relationships between sleep disturbance and severity of symptoms indicative of gastro-oesophageal reflux

The 67% of children with AS who scored above the clinical cut-off for behavioural symptoms indicative of gastro-oesophageal reflux (hereafter referred to as GORD symptoms) had significantly higher scores on the sleep-disordered breathing (*U* = 319.0, *p* = .005) and parasomnia subscales (U = 300.0, *p* = .003). There was a trend towards significance for higher daytime sleepiness scores for children who scored above the clinical cut-off for indicative GORD symptoms compared to children without clinical symptoms (366.0, *p* = .025).

Children with ASD who scored above the clinical cut-off for behavioural symptoms indicative of gastro-oesophageal reflux (hereafter referred to as GORD symptoms) had significantly higher scores on the night waking (*U* = 49.0, *p* = .009), parasomnia (*U* = 31.0, *p* = .001) and the sleep-disordered breathing subscales (*U* = 45.0, *p* = .009) than children with ASD who did not score above the cut-off. A trend towards significance was also found for children with ASD with GORD symptoms for increased daytime sleepiness (*U* = 53.0, *p* = .015).

Children with SMS with GORD symptoms had significantly higher sleep-disordered breathing scores (*U* = 8.5, *p* = .001). Children with TSC experiencing GORD symptoms had significantly higher scores on the sleep onset latency (*U* = 11.0, *p* = .005) and night waking scores (*U* = 11.0, *p* = .005) than children not meeting the cut-off for clinical GORD symptoms. A trend towards significance was also found for children with TSC with GORD symptoms for higher scores on the bedtime resistance (*U* = 16.5, *p* = .019) and parasomnia (*U* = 19.5, *p* = .037) subscales compared to children without GORD symptoms.

### Relationships between overactivity and impulsivity and sleep disturbance

The associations between sleep parameters and overactivity/impulsivity are presented in Table 5.

**Table 5:** Spearman’s correlations between overactivity and impulsivity and sleep quality

			Anxiety	Bedtime resistance	Sleep onset latency	Night waking	Sleep-disordered breathing	Parasomnias	Drowsiness
AS^a^	Overactivity	*r*_*s*_	–	.055	.179	.232	.308	.232	.332
	Impulsivity	*r*_*s*_	–	.323	− .077	.150	− .031	.181	− .145
SMS	Overactivity	*r*_*s*_	− .182	− .004	.433*	.034	.199	.443*	− .043
	Impulsivity	*r*_*s*_	.032	.059	.396*	.109	.350	.273	.478*
TSC	Overactivity	*r*_*s*_	.057	.329	.247	.725**	.576**	.628**	.477*
	Impulsivity	*r*_*s*_	.162	.480*	.262	.785**	.271	.362	.244
ASD	Overactivity	*r*_*s*_	− .060	.202	.435*	.249	− .089	.497**	.045
	Impulsivity	*r*_*s*_	.171	.318	.245	.266	− .009	.199	− .006

*p < .05, **p < .01

^a^Due to the majority of verbal items on the sleep anxiety subscale, children with AS were not included in this analysis

The data in Table 5 reveal that higher impulsivity subscale scores were moderately positively correlated with increased sleep onset latency and daytime drowsiness in children with SMS (both trend only, *p* < .05). Overactivity scores were associated with higher sleep onset latency and parasomnias scores in the SMS group (moderate associations, both trend only). Children with ASD’s sleep onset latency (trend only) and parasomnias scores were moderately correlated with the overactivity subscale score in a positive direction. Night waking and parasomnias scores were strongly positively correlated with the overactivity score in children with TSC.

Severity of sleep-disordered breathing and daytime drowsiness (trend only) were moderately correlated with overactivity scores in the TSC group. The impulsivity subscale score was significantly strongly positively correlated with increased night waking and was significantly positively correlated with the bedtime resistance score reflecting a moderate effect size in the TSC group (trend only).

### Relationship between sleep-disordered breathing and obesity (aim 4)

Sleep-disordered breathing scores for children classed as overweight, very overweight or severely obese were compared to those of children with a healthy weight. No significant differences in sleep-disordered breathing scores were found between healthy and overweight children with TSC, AS, SMS or ASD.

### Relationships between daytime drowsiness and sleep-disordered breathing (aim 5)

No significant relationships were found between daytime drowsiness and sleep-disordered breathing.

## Discussion

This study compared the profile of sleep disturbance across children with AS, SMS, TSC and ASD and compared the syndrome profiles to a chronologically age-matched TD contrast group. Children with AS, SMS, TSC and ASD had significantly higher night waking, parasomnia and daytime sleepiness scores compared to typically developing children; hence, there is evidence of broad vulnerability to sleep disturbance for children with neurodevelopmental disorders. Different profiles of sleep disturbance emerged when comparing different neurodevelopmental disorder groups, evidencing specificity of certain sleep disturbances in specific neurodevelopmental disorder groups; for example, sleep-disordered breathing and early morning waking were the most severe for children with SMS compared to other disorder groups. Children with ASD had a profile of sleep disturbance reflecting difficulties with sleep onset which was more severe than children with SMS, AS and TSC and typically developing children. The data in Table 6 highlight the profile of sleep-related disturbances compared to the TD group. The use of effect sizes enables the standardisation of results for each group compared to the TD group, to compare the relative severity of sleep disturbance for each group, despite the different sample sizes.

**Table 6:** Profiles of sleep disturbance in children with ASD, SMS, AS and TSC compared to TD children

	AS	SMS	TSC	ASD
Sleep anxiety	–	O	O	O
Bedtime resistance	O	O	O	+
Sleep onset latency	O	O	+	++
Night waking	++	++	+	+
Sleep-disordered breathing	+	++	O	+
Parasomnias	+	++	+	+
Daytime sleepiness	++	++	+	+
Severe early morning waking problems	++	+++	+	++

*O* effect size *r* < 0.2, + small effect size *r* 0.20–0.49 or odds ratio 0.2–9.0, ++ medium effect size *r* 0.5–0.8 or odds ratio 10.0–49.0, +++ odds ratio > 50

The evidence of daytime sleepiness or drowsiness, night time waking and parasomnias across the disorder groups as shown in Table 6 indicates that these sleep-related disturbances should be routinely assessed. The results of this study demonstrate that sleep disturbances are problematic in children with TSC, relative to typically developing children, even if no specific profile of sleep disturbance emerged compared to other disorder groups. These findings contrast with those of Hunt and Stores [5], who found children with TSC to experience settling in addition to night waking disturbance compared to a group of children with an intellectual disability of heterogeneous origin and typically developing children. Differences between the findings in the present study and the study by Hunt and Stores (1994) may be explained by differences in sample size; 20 parents of children with TSC completed the current study versus 40 parents in Hunt and Stores’ study. It is also possible that given the significant heterogeneity in TSC, children in the two studies may have differed in their degree of intellectual disability, as children with a moderate to severe level of intellectual disability in Hunt and Stores’ study had poorer overall sleep quality as measure by a sleep index score.

The profiles of sleep disturbance for children with ASD, SMS and AS delineated in the present study support and extend the current literature on sleep in these disorders. These data demonstrate that children with ASD predominantly experience difficulties with night waking and settling, children with SMS primarily experience night waking and early morning waking problems and children with AS primarily experience night waking difficulties, followed by early waking problems. The comparison of profiles of sleep disturbance across neurodevelopmental groups reveals that in some groups, sleep disturbance profiles affect the group homogenously, whilst in other groups, sleep disturbance severity and profile differs to a greater extent between children. This is apparent when comparing the frequency of severe sleep problems in children with SMS and AS. Half of the sample with AS did not experience any severe sleep problems, compared with only 8% of children with SMS. This is important when considering the specificity of sleep disturbance for each disorder group. It could be argued that early morning waking reflects partial specificity for SMS, as whilst 23% of children with ASD also experienced this problem, the majority (73%) of children with SMS experienced early morning waking. In contrast, severe night waking is less easily justified as reflecting partial specificity for children with AS, ASD and TSC as less than half of the children in each group experienced this disturbance. It could therefore be argued that children with AS, ASD and TSC are at higher risk of experiencing night waking, but it should not be considered a consistent feature of the phenotype of each disorder. Whereas 92% of children with SMS experienced some form of severe sleep disturbance, therefore, sleep disturbance could be argued to form part of the phenotype of SMS. Nonetheless, the presence of severe sleep disturbance for some children with AS, TSC and ASD means that clinicians must ensure that families are supported with appropriate assessment of and treatment for sleep disturbance where necessary.

No relationships between age and sleep disturbance were found for children with ASD, TSC and SMS. However, a negative relationship with age and night waking scores was found for the AS group. As the neurodevelopmental groups were well matched for chronological age, this suggests that the finding that night waking decreases with age in AS is a syndrome-specific characteristic, supporting previous research [11, 21]. It is also of clinical importance for families and professionals as this finding suggests that sleep disturbance may improve as children enter their teenage years. However, due to the cross-sectional nature of this study, this finding needs to be interpreted with caution and substantiated with a large-scale longitudinal study of sleep quality in children with AS.

The data from this study revealed that variation in sleep disturbance prevalence within the AS group could not be explained by differences between different genotypes of AS, although only small numbers of children without the *UBE3A* deletion genotype were included in the study. These findings reflect those of two out of three previous studies [11, 60, 61].

Children with ASD who were classed as non-verbal were more likely to experience early morning waking problems than children who were verbal (trend only). This could be attributed to children’s poorer ability to self-soothe, although lack of verbal ability cannot be substituted for poorer adaptive ability skills. This supports previous research [62]. No other associations between verbal ability or adaptive functioning and sleep parameters were identified for any group.

Mixed relationships were identified between sleep disturbances and neurological and health problems (see Table 7 for a summary of significant associations). No relationship between the experience of seizures in the last month and severity of sleep disturbance was found for either children with AS or children with TSC. This finding for children with AS was consistent with the existing mixed literature. However, it does not support a previous study in children with TSC [5]. Future research should use a validated questionnaire measure to assess seizure severity and correlate this with sleep disturbance, and should evaluate the relationship between nocturnal seizures and sleep disturbance on a night by night basis. For children with more health problems, in this study children with AS and TSC, a greater severity and number of health problems were associated with higher sleep-disordered breathing and parasomnias (TSC only). Symptoms indicative of GORD were associated with sleep-disordered breathing (AS, SMS and ASD groups) and parasomnias (AS and ASD groups). Children with symptoms indicative of GORD had significant higher night waking and sleep onset latency scores in the TSC group and higher night waking scores in the ASD group. However, as the questionnaire to assess GORD symptoms has not yet been validated, these findings should be interpreted with caution. These findings are supported by a recent study that demonstrated that children with ASD with gastrointestinal symptoms were significantly more likely to have interrupted sleep than children without gastrointestinal symptoms [63]. These findings demonstrate the importance of considering children’s broader health in relation to sleep quality, in particular with regard to mechanism between sleep-disordered breathing and GORD symptoms, as nocturnal arousals associated with gastro-oesophageal reflux have been detailed in previous research [64]. Greater sleep disturbance for children with AS or TSC with painful health conditions presents a clear first-line intervention for sleep disorders in children with neurodevelopmental disorders at risk of pain. These findings build upon the evidence of the importance of assessing and treating pain in children with neurodevelopmental disorders as a priority [65].

**Table 7:** Summary of significant (*p* < .01) associations between sleep disturbance, health conditions and behaviour for each group

	AS	SMS	TSC	ASD
Age	+ age/night waking	N/A	N/A	N/A
Health	+ number and severity of health conditions/sleep disordered breathing + symptoms indicative of GORD/sleep-disordered breathing/parasomnias	+ symptoms indicative of GORD/sleep-disordered breathing	+ number and severity of health conditions/sleep-disordered breathing/parasomnias + symptoms indicative of GORD/sleep onset latency/night waking	+ symptoms indicative of GORD/night waking/parasomnias/sleep-disordered breathing
Overactivity and impulsivity	N/A	N/A	+ overactivity and impulsivity/night waking + overactivity/parasomnias/ sleep-disordered breathing	+ overactivity/parasomnias

*N/A* no significant correlation, *p* > .01; + significant positive correlation

This is the first study, to the authors’ knowledge, to identify sleep-disordered breathing as a specific problem in children with SMS. The mechanisms underlying sleep-disordered breathing in this group remain unclear, as despite over a quarter of the sample of children with SMS having a BMI classified as overweight or obese, no significant difference in sleep-disordered breathing scores was found between these children and those who had a healthy weight or who were underweight. However, with missing data for BMI for 38% of the SMS sample, these data do need to be interpreted with caution. An alternative hypothesis is that the midface hypoplasia associated with SMS is associated with sleep-disordered breathing in this group. Midface hypoplasia, in which the mandibular (lower jaw) protrudes relative to the maxilla, affects over 90% of individuals with SMS and has been associated with obstructive sleep apnoea [66–68]. Similarly, for children with AS, despite obtaining higher sleep-disordered breathing scores than the TD contrast group, no relationship was found between BMI status and sleep-disordered breathing scores. Importantly, the AS group had complete BMI data for 94% of the sample. These data support findings from a previous questionnaire study that identified higher sleep-disordered breathing and snoring scores in children with AS compared to typically developing children [11]. However, it is important to note that the modified Simonds and Parraga sleep questionnaire used in the present study assessed sleep-disordered breathing based upon parental report. These scores are not interchangeable with a diagnosis of obstructive sleep apnoea which can only be assessed using polysomnography. Thus, further research is needed to assess the prevalence of obstructive sleep apnoea in children with SMS and AS using polysomnography.

No relationship was found between sleep-disordered breathing and daytime drowsiness, in contrast to literature cited in a recent systematic review [34]. However, the review only referred to two studies that had evaluated daytime sleepiness in the typically developing population objectively using the multiple sleep onset latency test. Therefore, further research should be conducted using a validated measure of daytime sleepiness in groups of children with neurodevelopmental disorders.

This was the first study to investigate the putative association between daytime sleepiness and overactivity and impulsivity in children with specific neurodevelopmental disorders. The emerging pattern of trend towards significant moderate correlations for two out of the four groups for overactivity or impulsivity and daytime drowsiness was an important finding. The results identified a positive correlation between overactivity and daytime drowsiness (trend only) and night waking for children with TSC. However, no relationships between sleep-disordered breathing and overactivity or impulsivity were found for children with SMS or AS, for whom the highest sleep-disordered breathing scores was identified. In addition, when the relationship between daytime drowsiness and sleep-disordered breathing was examined, no relationship for any of the neurodevelopmental disorder groups was found. Moreover, the sensitivity of both the drowsiness item and the sleep-disordered breathing subscale when investigating this relationship has not been established. Further research is needed to investigate the nature of the relationship between sleep-disordered breathing, overactivity and impulsivity and daytime sleepiness. This is particularly important to establish whether sleep-disordered breathing, which can be treated, impacts adversely children’s behaviour and daytime functioning.

### Limitations

This study provides the first broad overview of sleep disturbance in a cross-comparison study of four neurodevelopmental disorders compared to a typically developing sample. It is important to note that the neurodevelopmental groups comprise relatively small samples with wide age ranges from 2 to 15 years. Future studies should seek to evaluate whether the results reported here are replicable in larger samples and whether they are representative for children of differing ages. A broad categorisation of verbal ability, 30 words or signs were used to identify children as verbal or non-verbal in this study, which may have underestimated children’s ability to use non-verbal communication with caregivers. Future research should include a comprehensive measure to assess verbal and non-verbal communication and the relationship with sleep disturbance and include the assessment of IQ and adaptive functioning as reliable measures of ability.

## Conclusions

This study was the largest to date incorporating sleep disturbance questionnaire data from children with four distinct neurodevelopmental disorders using a measure designed for use with this population. This methodology has revealed broad vulnerability to sleep problems across neurodevelopmental disorders, such as night waking, but also partial specificity of sleep problems pertaining to early morning waking in SMS, and sleep-disordered breathing in children with SMS and AS. Risk profiles for sleep disturbances in each group were identified through the use of a typically developing contrast group. Whilst there were limitations to the evaluation of daytime sleepiness in this study, there are implications for the agenda for future research, particularly evaluating the relationship between sleep-disordered breathing and daytime sleepiness using polysomnography. These results provide further evidence for the importance of considering health issues in relation to sleep disturbance, as symptoms indicative of gastro-oesophageal reflux were associated with sleep-disordered breathing for the AS, SMS and ASD groups, whilst a greater number and severity of health conditions were associated with higher sleep-disordered breathing scores for children with AS and TSC. Taken together, these results enable delineation of syndrome-specific profiles of sleep disturbance in children with AS, SMS, TSC and ASD and have compared their severity to those experienced by TD children.

## Additional file

Additional file 1: **Table S1.** Between group contrasts on the MSPSQ to include *U* or *Chi-squared* statistics and *p* values. (DOCX 18 kb)
