# Supplementary material for: A cross-syndrome cohort comparison of sleep disturbance in children with Smith-Magenis syndrome, Angelman syndrome, autism spectrum disorder and tuberous sclerosis complex
Source: J Neurodev Disord. 2018 Mar 1;10:9. doi: 10.1186/s11689-018-9226-0 (PMC5831859; doi:10.1186/s11689-018-9226-0)
Supplement: Supplementary file 1 — Table S1. Between group contrasts on the MSPSQ to include U or Chi-squared statistics and p values. (DOCX 18 kb) [file 11689_2018_9226_MOESM1_ESM.docx]

|  | AS vs SMS | AS vs ASD | AS vs TSC | AS vs TD | SMS vs ASD | SMS vs TSC | SMS vs TD | ASD vs TSC | ASD vs TD | TSC vs TD |
| --- | --- | --- | --- | --- | --- | --- | --- | --- | --- | --- |
| Sleep anxiety | - | - | - | - | 327.0  P=.392 | 240.5  P=.661 | 542.0  P=.421 | 232.5  P=.232 | 563.5  P=.116 | 464.5  P=.939 |
| Bedtime resistance | 623.0  P=.033 | 621.0  P=.002* | 524.0  P=.086 | 1469.5  P=.426 | 294.0  P=.233 | 249.0  P=.982 | 446.0  P=.119 | 256.5  P=.494 | 449.0  P=.017 | 391.0  P=.334 |
| Sleep onset latency | 699.5  P=.064 | 446.0  P<.001* | 608.5  P=.346 | 1380.0  P=.236 | 122.0  P<.001* | 170.5  P=.038 | 513.5  P=.366 | 153.0  P=.003* | 215.5  P<.001* | 330.5  P=.074 |
| Night waking | 791.0  P=-.318 | 979.0  P=.590 | 563.5  P=.181 | 428.0  P<.001* | 319.0  P=.235 | 175.5  p=.055 | 54.5  P<.001* | 260.5  P=.431 | 272.5  P<.001* | 241.0  P=.005* |
| Sleep disordered breathing | 524.0  P=.006* | 877.0  P=.282 | 503.5  P=.053 | 697.5  P<.001* | 200.5  P=.008* | 107.5  p=.001* | 110.0  P<.001* | 249.5  P=.384 | 430.5  P=.011 | 359.0  P=.186 |
| Parasomnia | 565.0  P=.017 | 794.0  P=.089 | 658.5  P=-.686 | 751.0  P<.001* | 325.5  P=.687 | 155.5  P=.046 | 121.5  P<.001* | 227.5  P=.203 | 299.5  P<.001* | 242.0  P=.002* |
| Daytime sleepiness | 681.5  P=.054 | 862.5  P=.148 | 544.0  P=.121 | 521.5  P<.001* | 236.0  P=.009* | 145.0  P=.009* | 107.5  P<.001* | 287.0  P=.788 | 374.5  P<.001* | 260.0  P=.001* |
| Drowsy during the day | 370.5  p<.001* | 864.0  P=.129 | 559.0  P=.139 | 840.0  P<.001* | 114.0  P<.001* | 80.5  P<.001* | 94.0  P<.001* | 293.0  P=.867 | 503.5  P=.001* | 350.5  P=.004* |
| Co-sleeping | 348.5  P=.624 | 417.0  P=.589 | 264.0  P=.439 | 497.0  P=.011 | 371.0  P=.939 | 206.0  P=.270 | 458.5  P=.064 | 246.5  P=.237 | 567.0  P=.078 | 293.5  P=.004* |
| Severe night waking (%) | 9.42  P=.002* | .048  P=.826 | .003  P=955 | 23.45  P<.001* | 8.18  P=.004* | 6.38  P=.012 | 45.41  P<.001* | .014  P=.907 | 17.83  P<.001* | 16.97  P<.001* |
| Severe early morning waking (%) | 25.35  P<.001* | .30  P=.585 | .14  P=.713 | 7.21  P=.007* | 13.86  P<.001* | 15.28  P<.001* | 42.36  P<.001* | .52  P=.470 | 8.85  P=.003* | 4.14  P=.042 |
| Severe settling (%) | .446  P=.504 | 13.15  P<.001* | 2.87  P=.09 | <.001  P=.994 | 4.39  P=.036 | .623  P=.430 | .382  P=.537 | 1.48  P=.224 | 9.91  P=.002* | 2.35  P=.126 |

Supplementary table

*Between group contrasts on the MSPSQ to include U or Chi-squared statistics and p values*

Significant P<.01 *
